# NIFtHool: an informatics program for identification of NifH proteins using deep neural networks

**DOI:** 10.12688/f1000research.107925.1

**Published:** 2022-02-09

**Authors:** Jefferson Daniel Suquilanda-Pesántez, Evelyn Dayana Aguiar Salazar, Diego Almeida-Galárraga, Graciela Salum, Fernando Villalba-Meneses, Marco Esteban Gudiño Gomezjurado

**Affiliations:** 1 Escuela de Ciencias Biológicas e Ingeniería, Universidad de Investigación de Tecnología Experimental Yachay, Urcuquí, Imbabura, 100115, Ecuador

**Keywords:** Deep Neural Network, Embedding Layers, NifH protein, Software, k-mers

## Abstract

Atmospheric nitrogen fixation carried out by microorganisms has environmental and industrial importance, related to the increase of soil fertility and productivity. The present work proposes the development of a new high precision system that allows the recognition of amino acid sequences of the nitrogenase enzyme (NifH) as a promising way to improve the identification of diazotrophic bacteria. For this purpose, a database obtained from UniProt built a processed dataset formed by a set of 4911 and 4782 amino acid sequences of the NifH and non-NifH proteins respectively. Subsequently, the feature extraction was developed using two methodologies: (i) k-mers counting and (ii) embedding layers to obtain numerical vectors of the amino acid chains. Afterward, for the embedding layer, the data was crossed by an external trainable convolutional layer, which received a uniform matrix and applied convolution using filters to obtain the feature maps of the model. Finally, a deep neural network was used as the primary model to classify the amino acid sequences as NifH protein or not. Performance evaluation experiments were carried out, and the results revealed an accuracy of 96.4%, a sensitivity of 95.2%, and a specificity of 96.7%. Therefore, an amino acid sequence-based feature extraction method that uses a neural network to detect N-fixing organisms is proposed and implemented. NIFtHool is available from: https://nifthool.anvil.app/

## Introduction

Nitrogen is an essential nutrient for plants. Nitrogen fertilizers have the highest worldwide demand during agricultural practices. ^1^^,^^2^ Among the vast diversity of microorganisms, different bacterial taxa have developed the capacity to use atmospheric nitrogen as a substrate to produce ammonia (NH _4_) through the biological nitrogen fixation (BNF) process. ^3^ BNF is the most critical pathway of incorporating N-NH _4_ inside the biosphere. ^4^ Estimating that only 10% of the total nitrogen incomes proceed from atmospheric precipitation, the rest is through this biological process. ^5^

**Figure 1.**
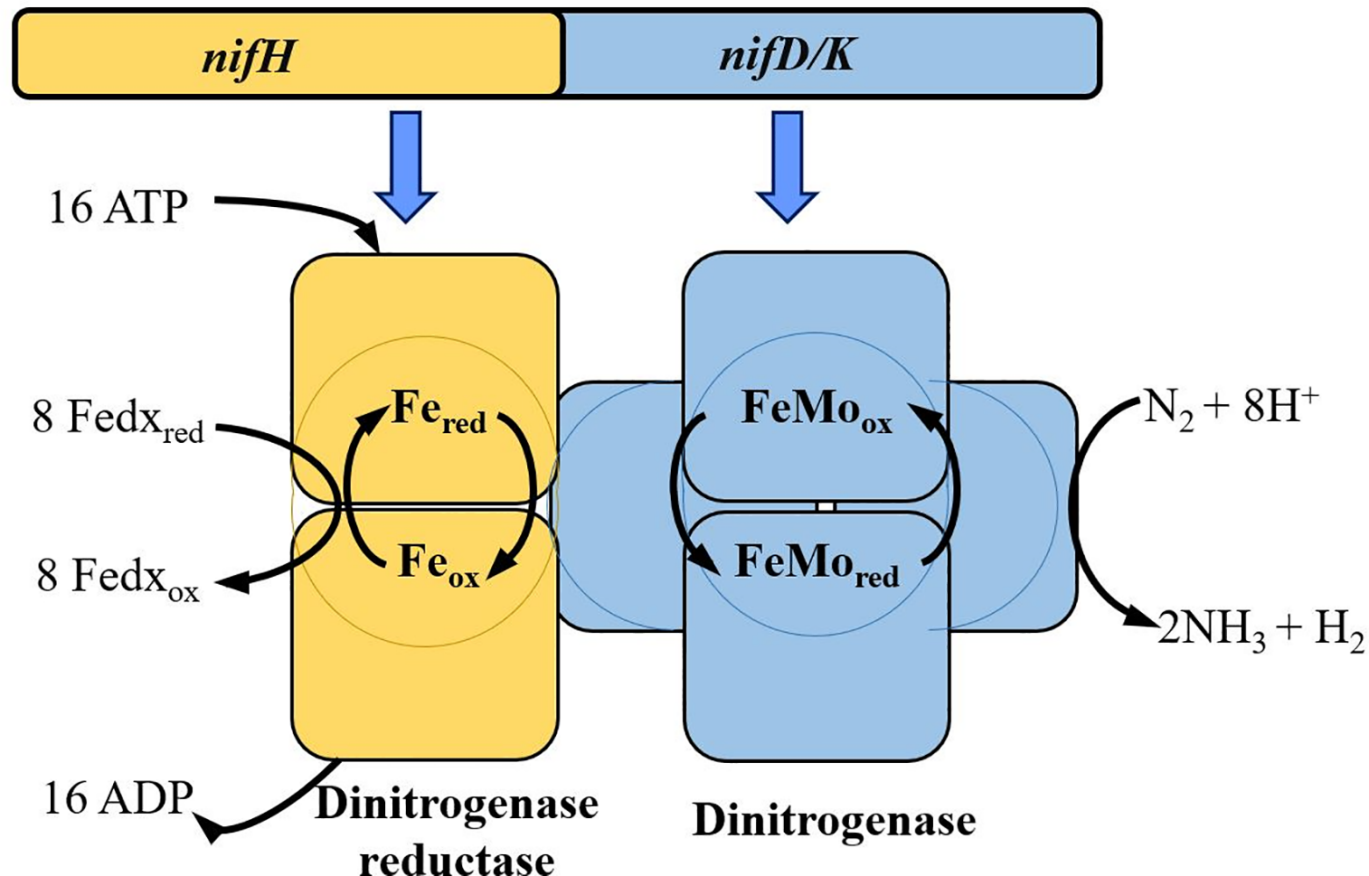
Nitrogenase complex. The *nifHDK* operon (genes encoding for the Fe/Mo-Fe nitrogenase protein complex) codifies for the subunits of the nitrogenase enzyme, which catalyzes the reduction of the N _2_ to NH _4_ in an ATP- dependent manner through the electron flux from the dinitrogenase reductase to the molybdenum-iron (Mo-Fe) protein subunit. Modified from Ref. 10.

The activity of the nitrogenase enzyme carry out the BNF. This enzyme is a molecular complex constituted by two subunits: (i) the dinitrogenase reductase (NifH), which is an iron protein that participates in the electrons transport from ferredoxins, to the (ii) dinitrogenase or molybdenum-iron-protein. ^3^^,^^6^ Molybdenum-iron-protein (Mo-Fe) is the catalytic site, which catalyses the N _2_ reduction using 16 ATP molecules as an energy source. ^7^ Both units are encoded in the *nifHDK* operon (genes encoding for the Fe/Mo-Fe nitrogenase protein complex) located on the bacterial chromosome or plasmids, depending on the bacterial species ^8^ ( Figure 1). The principal role of NifH protein is donating electrons to molybdenum-iron proteins (NifD/NifK), favouring N _2_ reduction. Efficient and quick identification of NifH proteins is of relevant interest because Nitrogen engineering focuses on the development of new products during farming activities. ^9^

Previous studies have focused on developing new informatics tools to identify *nifH* genes from different bacterial genera. Frank *et al*. (2016) retrieved the sequences of the *nifH* genes in the genome of nitrogen-fixing microorganisms and achieved the classification of the nifH sequence at different clusters. ^11^ Likewise, Shinde *et al*. (2019) developed his *nifH* genes classification model based on image processing and convolutional neural network. ^12^ On the other hand, Frank (2014) designed a tool with sufficient information to carry out phylogenetic cluster membership predictions from 32954 NifH protein sequences. ^13^ These three studies obtained good results. However, their investigations did not produce a computer tool.

Meher *et al*. (2018) developed nifPred, a machine learning (ML) software, to perform a sequence classification into NifH or non-NifH proteins. This informatics tool converts multi-categorical sort of gene sequences into one of the six types of the Nif proteins encoded by the *nif* operon using a high computational performance. ^14^

Constant supervision is necessary to guide the program in all phases of the system, which causes the increment of computational cost. ^15^ Nowadays, some algorithms are registered in literature to make predictions of NifH proteins based on gene sequences. However, there is still no tool to distinguish Nif proteins from amino acids sequences.

Based on this background, the objective of this work was to develop an informatics tool that uses deep neural networks with the lowest computer cost to play an essential role in the improvement of the BNF process research through the identification of the NifH proteins among different bacterial genera in a reliable way.

## Methods

**Figure 2.**
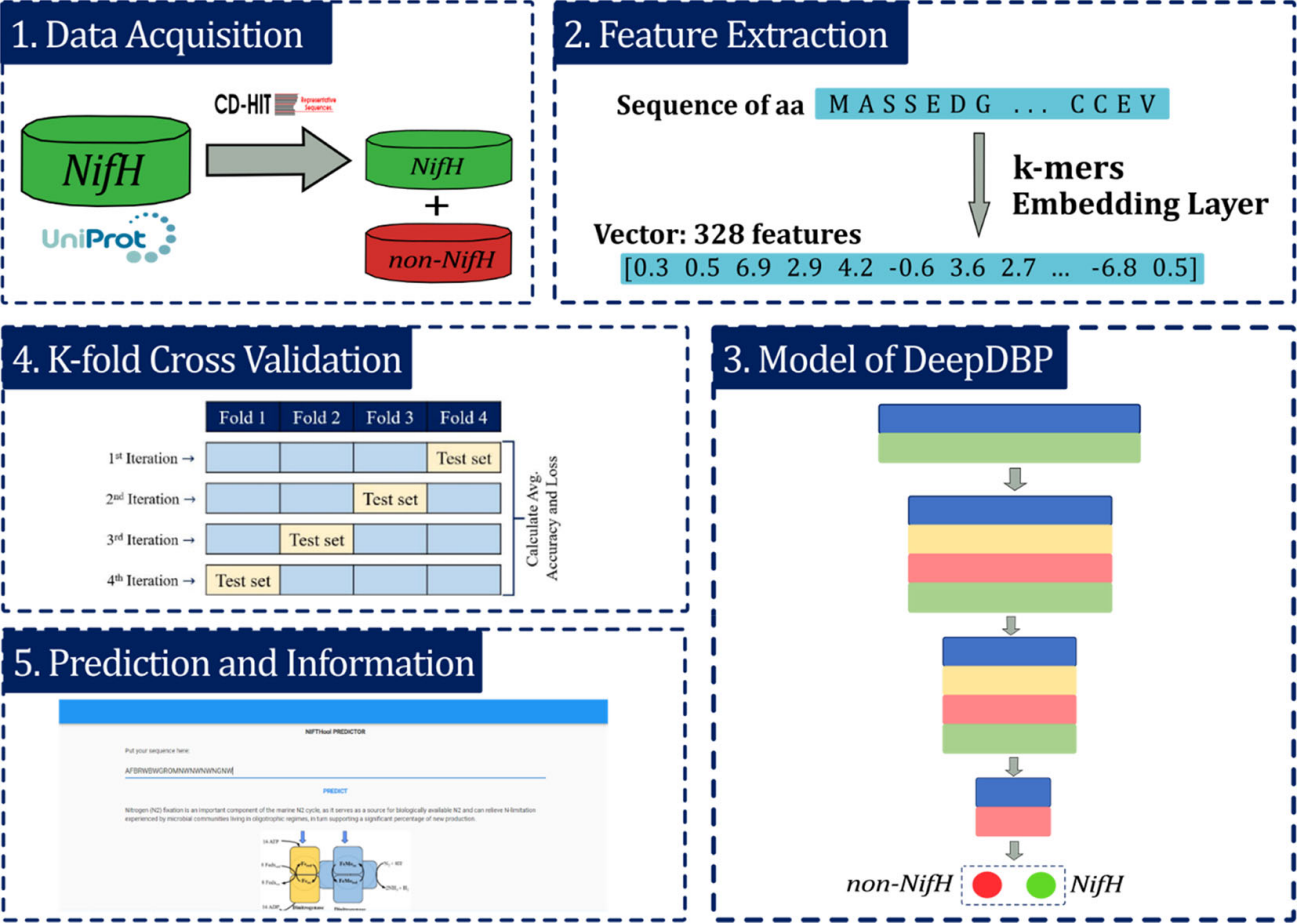
Description of the methodology applied in this work. i) Data acquisition, ii) Feature extraction, iii) Modeling of deep learning, iv) K-fold Cross-validation and v) prediction and providing information. Dinitrogenase reductase = NifH.

The proposed model was developed into five main stages: (i) acquisition of raw data from the UniProt protein databank ^16^ (Universal Protein Resource, RRID:SCR_002380); (ii) feature extraction stage, which allows defining numerical vectors as representation of amino acids (aa) sequences; (iii) development of a prediction model or classifier using a deep neural network (DNN); (iv) K-fold Cross-validation to evaluate the model and (v) the identification of the predicted label of the sequence ( Figure 2).

Experimental processes were carried out using an Intel Core i5-8100 processor machine, feature extraction process and neural network code were written in Python v. 3.8 language (RRID:SCR_008394), and the classifier was implemented on Anvil Works. ^17^

### Acquisition and preprocessing of raw data

The binary classification of the protein sequences was arranged under two types of labels: 1 (NifH) and 0 (non-NifH). The raw dataset was constructed by NifH and non_NifH protein sequences extracted from database UniProt ^18^ in the FASTA format up to March 10 ^th^ 2021. ^18^ NifH and non-NifH sequences were retrieved by searching for ‘NifH, nifH proteins’ and ‘non-NifH, non-nifH proteins’, respectively, considering the following parameters: organism identification, gen name, proteins names, and length. Uniprot provided 52942 NifH proteins and 5763 non-NifH at the end of the search.

**Figure 3.**
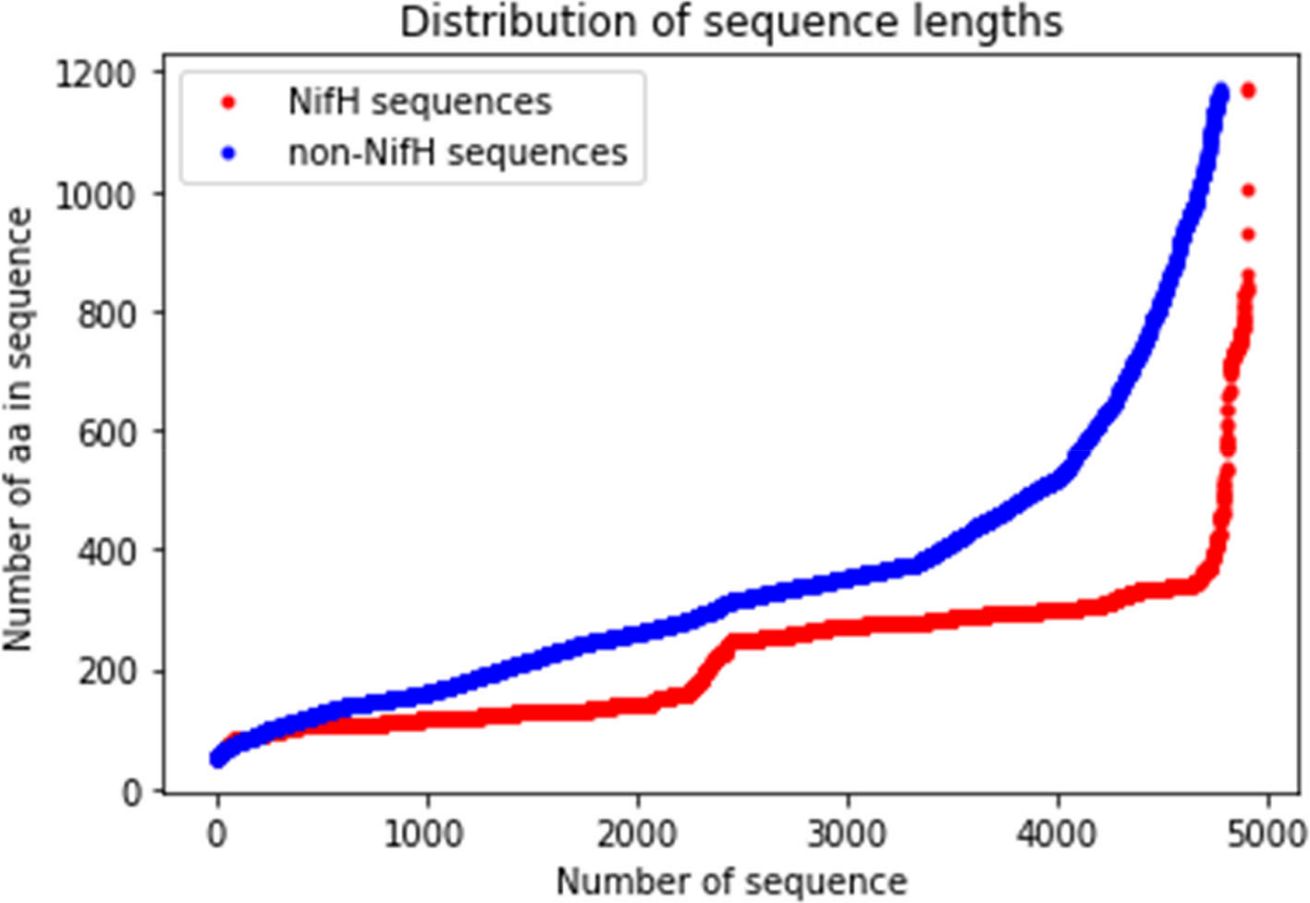
Correlation of the sequences number with the number of the amino acid (aa). The curves show the correlation between the number of sequences and the number of amino acids of dinitrogenase reductase (NifH) and non-NifH proteins.

CD-HIT software (RRID:SCR_007105) analysed the raw dataset ^19^ to remove redundant sequences with 90% similarity to avoid undesirable biases. Next, positive, and negative sequences, 4939 and 4953, respectively, were obtained after the cleaning process. Sequences were filtered for lengths greater than 50 aa and shorter than 1173 aa, the maximum length of NifH sequence. These values were set up for two reasons: (i) the upper limit because non-nifH sequences are greater than 1173 aa and (ii) shorter sequences than a defined upper limit are padded with zeros until the limit is reached that guarantees the conversion of aa sequences into numeric vectors during the embedding layers (feature extraction stage). For instance, for a maximum value defined as 2000, a 125 aa sequence would have to be completed with 1875 zeros; for a sequence of 1750 aa, it would only be necessary to complete it with 250 zeros. The redundancy of a large amount of zeros can be a factor leading to undesirable bias. ^20^ Thus, 4911 NifH and 4782 non-NifH protein sequences were obtained after filtration of limits to build the final dataset of 9693 sequences ( Figure 3).

### Feature extraction

This step results in 328-feature numeric vectors representing each of the sequences. The values were obtained through two different processes, k-mers, which are related to the presence of specific groups, and embedding vectors, related to the location of the amino acids in the sequence.

*k-mers*

**Figure 4.**
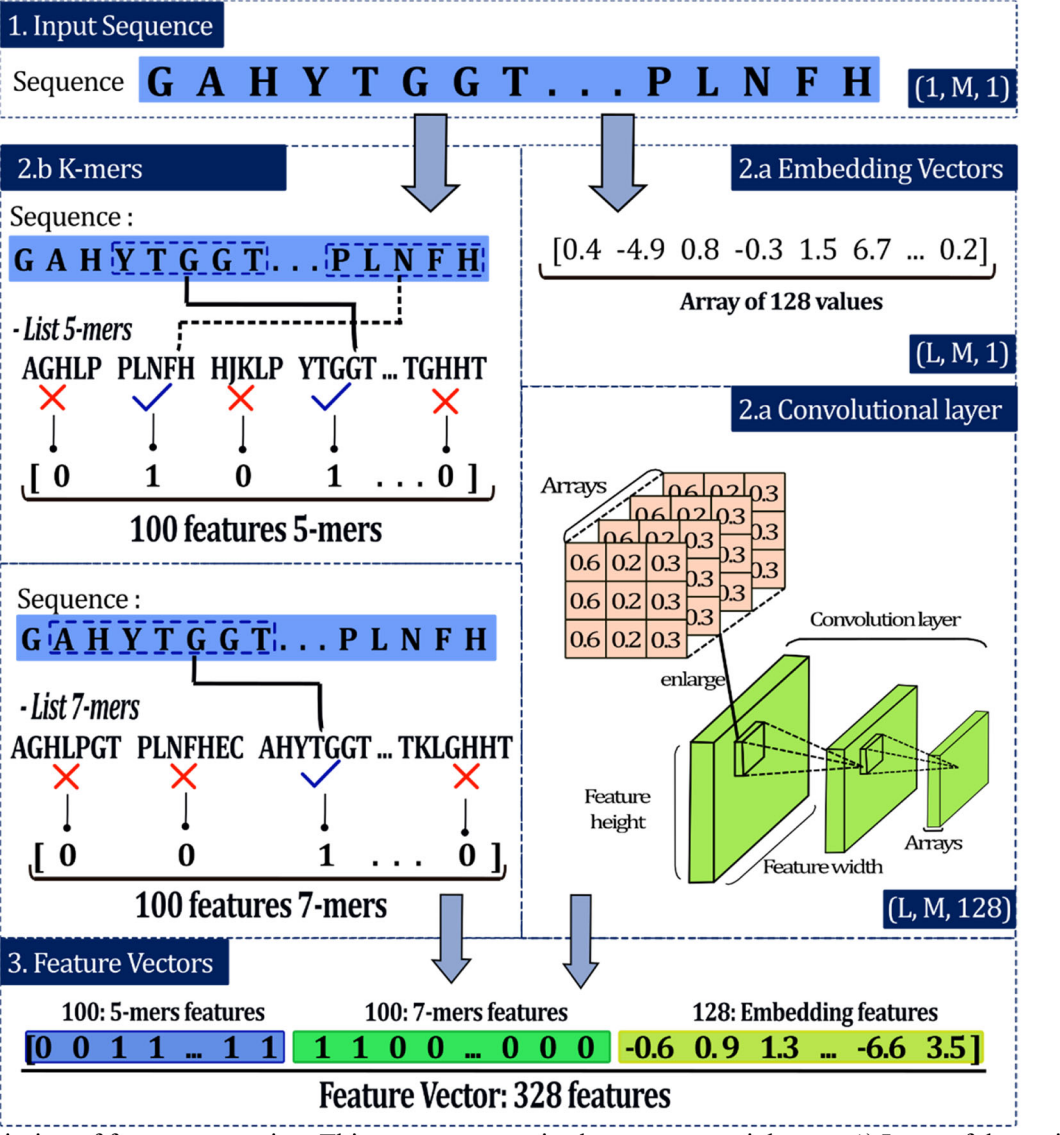
Description of feature extraction. This process comprised some sequential steps: 1) Input of the amino acid (aa) sequences, 2) extraction, and 3) development of the convolutional layer. Where M is the length of the input sequence and L is the length of the embedding vector.

This analysis allows the sequence representation based on the presence of specific groups called k-mers, where k is the length of that group. ^21^ For example, 5-mers represents a specific group of 5 amino acids. This stage begins with the generation of a list of the most common k-mers within the NifH dataset. Considering 5-mers, for each sequence of the dataset of 4939 NifH proteins, all the different 5-mers are obtained and their quantities are counted. Then, all the 5-mers in the entire dataset are identified and the k-mers with the highest frequency are defined, which form the general list (GL) of 5-mers where the order of each value is relevant. To generate a numerical vector for a certain sequence, GL is compared with that sequence and according to the presence of the GL 5-mers in the sequence, ‘1’ is recorded for presence and ‘0’ for absence according to the order of the GL ^22^ ( Figure 4).

For instance, in the sequence ‘GAHYTGGTPLNFH’ nine 5-mers can be identified, some examples of the 5-mers are GAHYT, AHYTG, HYTGG, PLNFH. If the GL of 5-mers were made up of 5 k-mers, for example: AGHLP, PLNFH, HJKLP, YTGGT and TGHHT the resulting vector for the example sequence would be [0, 1, 0, 1, 0]. The length of the vector is five because the length of the GL is five. According to the order, each GL 5-mer is searched in the sequence and if the 5-mer is present, 1 is recorded. In the example, the vector has ‘1’ at position 2 because PLNFH is present in the sequence and is the second most common of the GL.

*Embedding features*

This section was performed as described previously by Shadab *et al*. (2020) and comprised the carrying on: (i) input sequences, (ii) embedding vectors, and (iii) convolutional layers ^23^ ( Figure 4).

*Input sequences*

Protein sequences have different lengths depending on the number of aa. Deep learning requires the same length for all sequences. ^24^^,^^25^ The maximum size determined from the data had a value of 1173. Each number sequence with a length less than this value goes through a filling process, and the sequence was filled with the number “0” (token) until the maximum size is achieved ( Figure 4). These zero vectors do not affect the output of the subsequent layers, and M was defined as the length of the input sequence. ^23^^,^^24^ In addition, the aa count per chain was performed, and this set presented a minimum of 50, a maximum of 1173, and an average of 278.43 ( Figure 3).

*Embedding vectors*

Padded data were passed throughout an embedding layer to get a dense vector. This layer is used to transform discrete inputs into points in a vector space, called embedding vectors (L is the length of this vector). ^23^ Each aa of the protein sequences, both in the training and test sets, had a specific integer number, and the result of the encoding phase is the integer number vectors. For instance, in the sequence ‘ACLKIGAL’, a possible encoding would be ‘1-4-6-8-13-15-1-6’ ( Figure 4). This algorithm randomly assigns the digits but maintains the same number for a specific aa. The order in which the numbers are assigned could be a relevant aspect regarding the model's performance. However, it has been proved that order does not influence the result. ^23^^,^^25^ The final output of the embedding layers is a uniform matrix of size L×M.

*Convolutional layer*

A trainable convolutional layer was added, and its input was a uniform matrix (embedding vectors). This layer applied convolution using 128 trainable filters, each with a size of L×31. The result was 128 feature maps, each of the same size. To reduce overfitting and capture noise, we used a max-pooling (window size of 3×3) to subsample these feature maps. ^23^ Finally, this feature map was flattened into a 1×1 dimensional matrix, where each matrix represents the features of the input sequence ( Figure 4). These features were used to train the classifiers.

The advantage of this method relies on the results of the classification model that can be back-propagated to the convolution and embedding layers. ^23^^,^^25^^,^^26^ These layers were trained to extract better features.

### Dataset methodologies

This stage allows the determination of the the most efficient dataset to perform the training and evaluation of the neural network. For this purpose, different methodologies are used to create the datasets. Two techniques for feature extraction were considered, the methodologies will focus on the combination of these techniques and the number of features that are extracted. Due to the embedding vectors (EV) has 128 fixed features, the variation of dataset methodologies depends on the length of k-mers and number of k-mers in the GL. Some datasets were EV, 3-mers (100 features) + EV, 7-mers (300 features) + EV, 3-mers (100 f) + 5-mers (100 f) + EV, and so on. Some datasets were created by combining k-mers and EV, but others were created by combining two different k-mers and EV. Table 2 shows the different methodologies analysed. After a series of analysis with the dataset methodologies to determine the most optimal number of features to compose the numeric vectors the feature extraction stage results in vectors of 328 numeric values corresponding to 100 5-mer features, 100 features of 7-mers, and 128 values of embedding vectors ( Figure 4).

### Deep neural network

**Figure 5.**
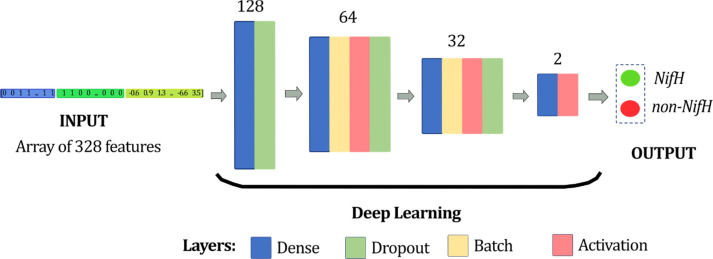
Visualisation of the deep neural network architecture. It was composed of four blocks, and the number of neurons for each block was 128, 64, 32, and 2, from the first to last one, respectively.

For the prediction of the identity of the aa sequence, a Deep Neural Network (DNN) was designed. Its input corresponds to the representation of the sequences (array of 328 numbers) and the output is the class of each sequence: 1 (NifH) and 0 (non-NifH). This DNN was written in Python v.3.8 using the following libraries: i) Pandas (RRID:SCR_018214), ^27^ ii) Keras, ^28^ iii) Scikit-learn (RRID:SCR_002577), ^29^ iv) NumPy (RRID:SCR_008633), ^30^ and v) Matplolib. ^31^ First, the hyperparameters of our model were related to the learning algorithm level: training of 40 epochs at 6 seconds, using a batch size of 40 and a learning rate of 6x10-5. Second, hyperparameters related to structure and topology were the layers. The deep learning model consisted of 12 layers, excluding the input and output layers ( Figure 5).

The model established the number of neurons in each defined block of layers, being 128 neurons for the first two layers, 64 neurons for the following four layers, followed by 32 neurons for the next four layers, and finally, two neurons at the last two. The number of neurons was placed according to the input parameters and the architecture of the DNN. ^32^ There were four layers in the neural network corresponding to the dense, activation, dropout, and batch normalization layers. ^26^^,^^33^ Four layers were used: (i) four dense layers, (ii) three activation layers, (iii) three dropout Layer, and (iv) two batch normalisation layers. The detailed configuration and order of layers of the proposed DNN model are shown in Table 1.

**Table 1.** Layers of the deep neural network implemented in this model.

Layer	Type	Output shape	Param #
dense_108	Dense	(None, 128)	42112
dropout_96	Dropout	(None, 128)	0
dense_109	Dense	(None, 64)	8256
batch_normalization_56	Batch	(None, 64)	256
activation_82	Activation	(None, 64)	0
dropout_83	Dropout	(None, 64)	0
dense_110	Dense	(None, 32)	2080
batch_normalization_57	Batch	(None, 32)	128
activation_83	Activation	(None, 32)	0
dropout_84	Dropout	(None, 32)	0
dense_111	Dense	(None, 2)	66
activation_84	Activation	(None, 2)	0

### k-fold cross validation

**Figure 6.**
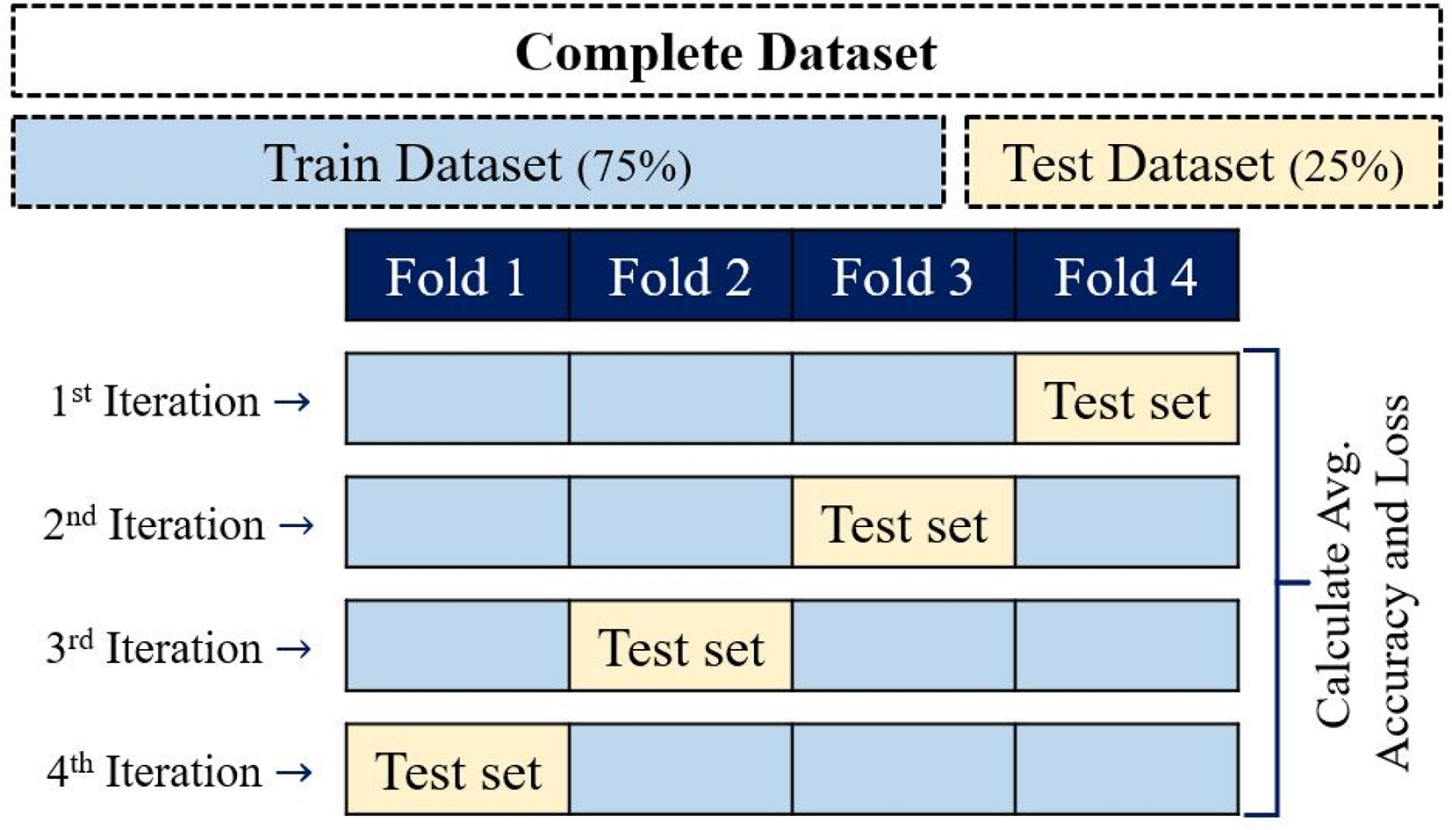
Four-fold cross-validation diagram. Data were divided into four folders, three were used to train the classifier, and one was allowed to test during each of four iterations.

To evaluate the classification accuracy of the several dataset methodologies considered to train the neural network ( Table 2), the k-fold cross-validation technique was performed. k-fold cross-validation divides datasets into k-subsets and requires that each subset is used to validate data exactly once. ^34^ In this validation process, k typically is 10, even though to reduce the computational time, this study used k = 4, giving 4-fold cross-validation ( Figure 6).

**Table 2.** Performance metrics (precision, recall, F1-score, accuracy, and loss) calculated for each dataset methodology.

Methodology	Statistical parameters (%)				
	Precision	Recall	F1-score	Accuracy	Loss
Embedding Vectors (EV)	93	93	93	92.82	20.96
3-mers (100 features) + EV	95	96	95	95.46	17.34
3-mers (200 features) + EV	96	96	96	95.58	21.42
3-mers (300 features) + EV	96	96	96	95.71	19.32
5-mers (100 features) + EV	95	95	95	95.38	13.54
7-mers (100 features) + EV	96	96	96	95.5	13.51
7-mers (200 features) + EV	95	95	95	95.38	16.14
7-mers (300 features) + EV	96	96	96	95.38	15.43
15-mers (100 features) + EV	95	95	95	94.97	15.45
15-mers (200 features) + EV	94	94	94	94.39	16.71
20-mers (100 features) + EV	94	94	94	93.69	18.96
20-mers (200 features) + EV	93	93	93	93.48	19.12
3-mers (100 f) + 5-mers (100 f) + EV	96	96	96	95.71	19.17
3-mers (100 f) + 7-mers (300 f) + EV	96	96	96	96.04	16.78
3-mers (300 f) + 7-mers (300 f) + EV	96	96	96	96.29	20.31
5-mers (100 f) + 7-mers (100 f) + EV	96	96	96	96.37	14.79
5-mers (100 f) + 7-mers (300 f) + EV	96	96	96	95.63	13.9

Datasets were partitioned into four equal parts; one group was used to test the methodologies (test dataset), and the remaining groups were used to train the software (Train Dataset). Four iterations were needed for each part to validate the methodologies throughout an accuracy and confusion matrix obtained for each iteration to discard the model. Then, each dataset methodology is processed by 4 iterations, where induvial iteration generates a classifier. Thus, when the classifier with the best performance is found, this is saved and used to predict NifH proteins.

### Operation

NIFtHool requires access to internet and to have any device capable of being a Web server. The device must have an operating system that can run as a Web server, capable of delivering HTML5 content. It must also have an Intel ^®^ Celeron ^®^ 847 Processor, 1.10 GHz, and a minimum Ram of 512 MB. Finally, this tool does not require the device to have a certain amount of storage or hard disk space as it works online.

## Results

### Evaluation of the model classification

Performance of four-fold cross-validation and metrics such as accuracy, loss, F1-score, sensitivity or recall, and precision were obtained for each dataset methodology to evaluate the classification as summarized in Table 2. Due to each dataset methodology works with four iteration producing four classifiers, only the one with the best performance is mentioned below.

The use of the methodology that consists of EV had a high performance, since both its precision, recall and F1-score were greater than 90%. However, its loss still represents a significant value. For this reason, EV was merged with k-mers to increase the classification values. A large number of methodological datasets were tested, and all of them were higher than the EV. The results define that datasets with longer k-mers, such as 20-mers, have slightly lower performance than datasets with shorter k-mers, such as 7-mers. Because the computational cost is higher as the k-mers are longer, and because their performance decreases slightly, shorter k-mers were selected to improve the classification performance.

Another factor considered when comparing the different methodologies was the number of features extracted. For the 3-mers + EV and 7-mers + EV datasets, the extraction of 100, 200 and 300 features were tested, but the results were similar between the groups. In the group of 3-mers + EV, the accuracy values were around 95% for the three cases, as well as recall and F1-score, which were the same (96%) for all. For the group of 7-mers + EV, despite being k-mers of greater length than the 3-mers, they obtained similar results to 3-mers + EV, where their greatest difference was the loss, which represents an advantage for the 7-mers with values between 13 and 17%, compared to the higher 17 to 21% of the 3-mers. In this sense, since a greater number of features implies a higher computational cost, and the values are similar, the extraction of 100 features for each k-mer was chosen. The analysis of 5-mers + EV was also recorded, which was similar to 3-mers + EV, however this analysis showed better performance in the loss, since 5-mers reached 13% while the best result of 3-mers was 17%.

Methodologies that combine two k-mers + EV were experimented with, and due to the high performance of each k-mer together with EV, the performance of the combination of these k-mers with EV was analysed. Finally, five dataset methodologies were performed, and 5-mers (100 features) + 7-mers (100 features) + EV dataset had the best performance, which obtained precision, recall and F1-scores of 96%, an accuracy of 96.37%, and a loss of only 14.79%. Due to its performance, this methodology was selected to be used as the model classifier and to be implemented into an informatics tool.

**Figure 7.**
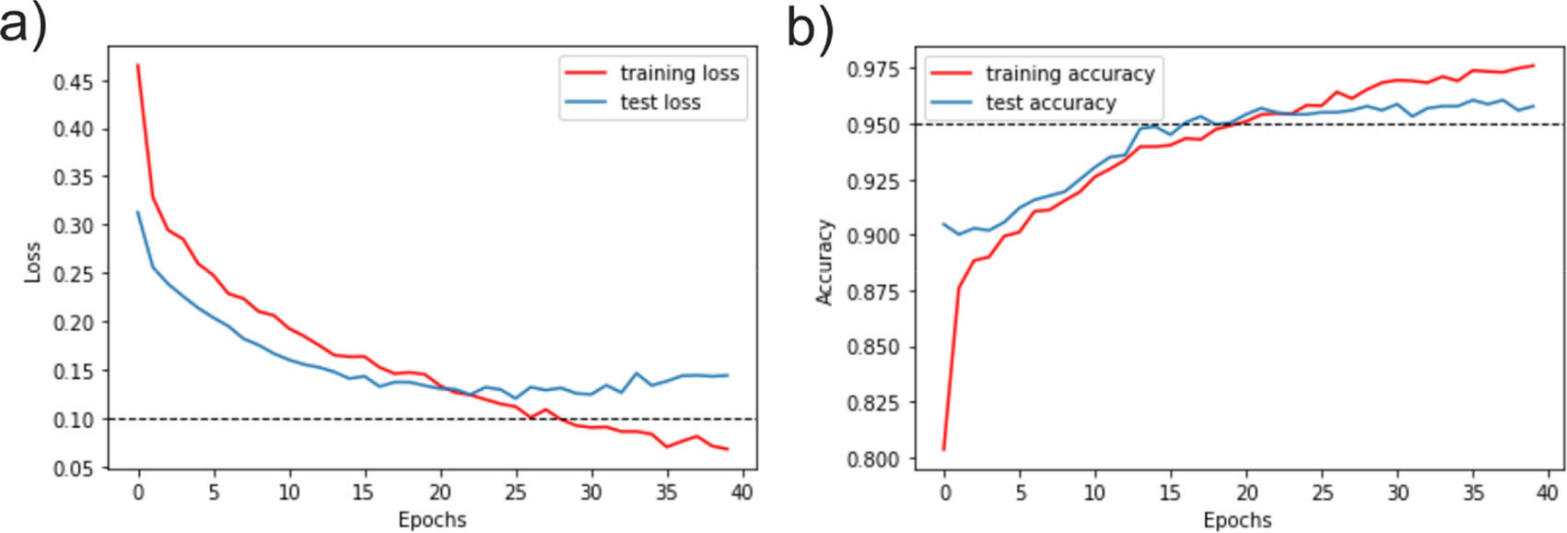
TensorBoard (RRID:SCR_016345) visualization of the distributed training metrics for the classifier after 30 epochs. Where x-axis represent the number of epochs and the y-axis represents the values of accuracy and loss as a function of unity (1= 100%). a) Loss evaluation. b) Accuracy evaluation.

The validation error of model 2 was 0.2625. This value was obtained from the learning rate, ranging from 0 to 40 in which the red epochs were trained. Measurements showed suitable training convergences as shown in Figure 7. Accuracy evaluation of the training started with low values that increased in the epochs ( Figure 7b). On the other hand, while training began with a high loss, this value decreases as they were trained ( Figure 7a). Both graphs show similar behaviour during the training and validation as a reliable model learning with constant values obtained at the end of the assessment, which indicates that the maximum training point was reached.

**Figure 8.**
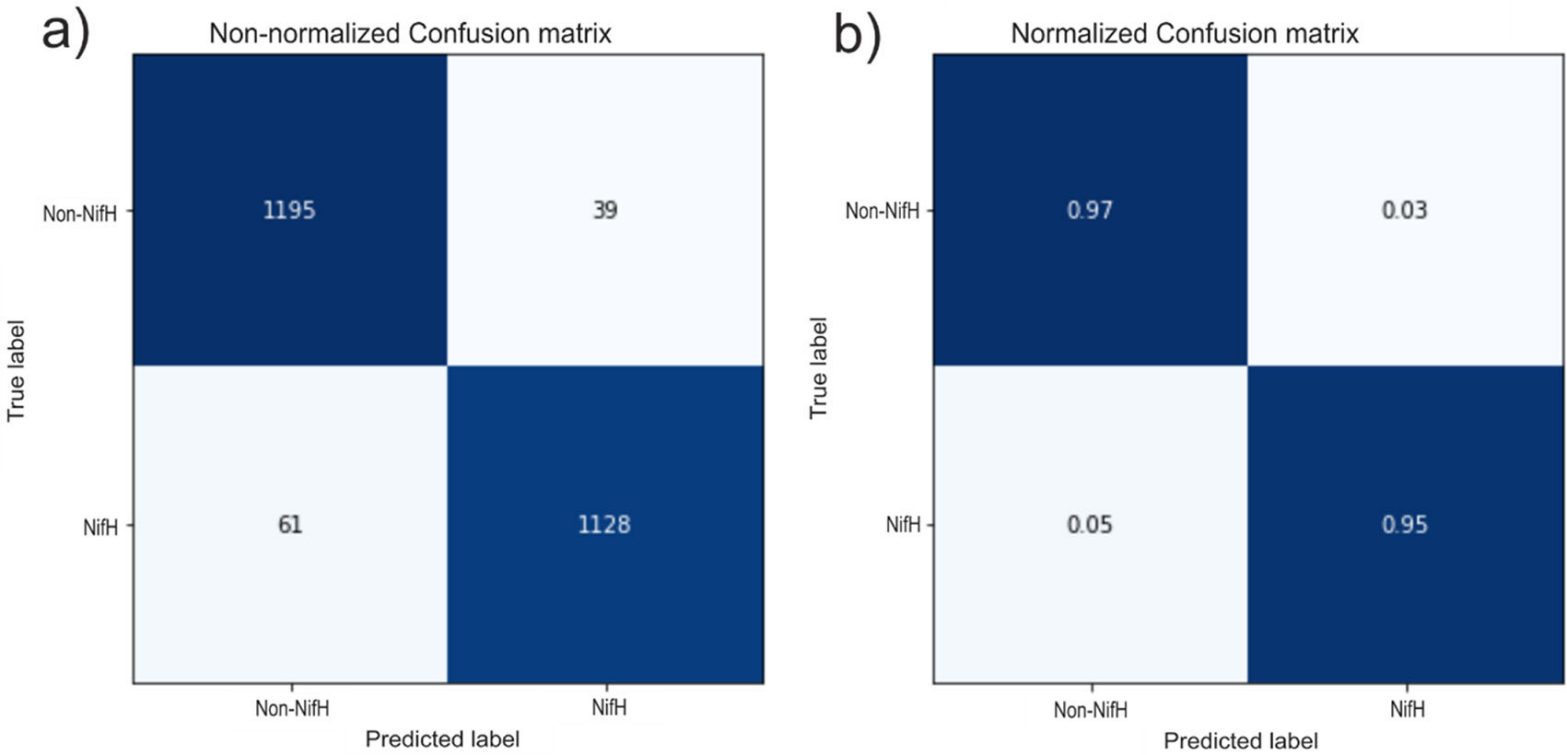
Assessment of the efficiency of the Deep Neural Network by a Confusion matrix. a) Panel a shows the confusion matrix for the number of evaluated sequences, and panel b corresponds to the number of evaluated sequences normalized to one. TP: true positive =1188, TN: true negative = 1132, FP: false positive = 25, and FN: false negative = 48.

The efficiency of the neural network was assessed using a confusion matrix. Primary diagonal data was represented, which indicates the number of hits in the model ( Figure 8). 1195 sequences were correctly classified as no-NifH, and 1128 as true NifH proteins. The value below the primary diagonal shows the false negatives or type II errors (the NifH is not detected), corresponding to 61 cases.

In contrast, the value above the primary diagonal reflected the classifier errors: false positives or error type I (the NifH is detected but not present) was equal to 39 cases. The confusion matrix results evaluated the relevance through 3 metrics: accuracy rate (96.4%), specificity (96.7%), and sensitivity (95.2%).

## Discussion

Deep learning was selected because it worked with protein sequence and conversion to arrays allowing better results than other methods, as previously reported ( Table 3). Our model had a high performance, as demonstrated high sensitivity (95.2%), high accuracy (96.4%), and specificity (96.7%), which is comparable to current deep learning techniques generating software ^23^^,^^25^ that considered a vector of 128 features-values for each sequence protein to train the model.

**Table 3.** Performance of our model in comparisons with other methods of machine learning.

Method	Learning technology	Purpose	Database	Metrics evaluated (%)			Reference
				Sensitivity	Specificity	Accuracy	
Embedding vectors and ^§^ANN	*NN	Identification of ^€^NifH proteins	9793	95.17	96.67	96.37	This work
Image processing and ^¶^CNN	NN	Identification of NifH proteins	42767	98.26	88.79	99.00	^12^
Feature Generation and ^¥^SVM	**ML	Identification of Nif proteins: NifH, ^γ^NifD, ^ɑ^NifK, ^∞^NifE, ^∂^NifN and ^ℇ^NifB.	747	88.70	99.30	94.00	^14^
Embedding vectors and ^£^MBD-LSTM	NN	Identification of *Plasmodium falciparum* mitochondrial proteins	3776	100	99.33	99.50	^25^
Embedding vectors and ^Þ^DeepDBP-ANN	NN	Identification of DNA-binding proteins	1261	98.00	97.00	99.02	^23^
^†^CART	ML	Classification of NifH Protein Sequences	32954	N/D	N/D	95-99	^13^
CART and decision trees	ML	Classification of NifH Protein Sequences	290	N/D	N/D	96-97	^11^

^§^ ANN: Artificial neural network.

^¶^ CNN: Convolutional neural network.

^¥^ SVM: Support vector machine.

^£^ MBD-LSTM: Multilayer bi-directional long short term memory.

^Þ^ DeepDBP-ANN: Deep neural networks for identification of DNA binding proteins.

^†^ CART: Classification and regression trees statistical models.

* NN: Neural networks.

** ML: Machine leaning.

^€^ NifH: Nitrogenase Iron Protein.

^γ^ NifD: Nitrogenase molybdenum-iron protein alpha chain.

^ɑ^ NifK: Nitrogenase molybdenum-iron protein beta chain.

^∞^ NifE: Nitrogenase iron-molybdenum cofactor biosynthesis protein NifE.

^∂^ NifN: Nitrogenase iron-molybdenum cofactor biosynthesis protein NifN.

^ℇ^ NifB: Nitrogenase iron-molybdenum cofactor biosynthesis protein NifB, N/D: No data.

Our software showed a high performance as compared with previous apps. For instance, Shinde's model ^12^ is based on the analysis of gene sequences of NifH proteins, operated with a 32×32 matrix for each sequence as our model. Nonetheless, our software is an improvement on previous informatics tools as it uses other feature extraction methods, as described above. nifPred, a multi NifH proteins classifier, that uses 13,500 values per sequence, involves four manual methods to obtain the components and data to be trained, having a high specificity. ^14^

NIFtHool was compared with two models that work with two different embedding vectors to identification of mitochondrial proteins of *Plasmodium falciparum* ^25^ and DNA-binding proteins. ^23^ The three models showed positive results in sensitivity, specificity, and accuracy, so the selection of the embedding vector method was adequate. Additionally, a comparison was made with studies using machine learning techniques for the classification of NifH protein sequences. These studies used classification and regression trees statistical models and decision trees. ^11^^,^^13^

It is stated that our model obtained high accuracy results like these models but with reduced computational power. Thus, NIFtHool shows clear improvements compared to the studies in the literature as shown in Table 3.

## Conclusion

A binary classification model of NifH protein sequences using artificial neural networks has been developed in the present work and hosted by Anvil. We tried the conventional approach of extracting features with specified algorithms through the novel feature extraction approach using deep learning techniques. Numerical features were obtained from two aspects: k-mers method considering the unique k-mer and an embedding layer related to aa position in the sequence. This methodology that was studied offers a performance similar to the best performances in the literature. Our tool offers better computational performance due to the classification process being based on the use of only NifH protein domain, resulting in less data processing for the software.

Even though the entire nitrogenase complex is relevant for transforming atmospheric nitrogen into ammonia, only NifH protein has been considered because this subunit is paramount during the reduction from N _2_ to NH _4_. NIFtHool not only represents a significant improvement compared to other computational methods, but it is also a tool for the identification of NifH Protein. Thus, researchers can easily use NIFtHool to identify NifH proteins as a reliable tool during the protein and nitrogen fixing bacteria analysis.

## Data availability

### Underlying data

Zenodo: NIFTHool: Repository. https://doi.org/10.5281/zenodo.5913032. ^18^

This project contains the following underlying data:

- List_kmers.csv (List of 5-mers and 7-mers obtained from dataset after it filtered sequences shorter than 50 aa and longer than 1173 aa)
- RAW_data_NifH.fasta (52942 NifH proteins retrieved from Uniprot)
- data_NifH.csv (4939 NifH sequences retrieved after CD-hit filtration)
- data_nonNifH.txt (4953 non-NifH sequences)

### Extended data

Zenodo: NIFTHool: Repository. https://doi.org/10.5281/zenodo.5913032. ^18^

This project contains the following underlying data:

- data_NifH_plus_nonNifH.txt (Combination of sequences from data_NifH.csv and data_nonNifH.txt).

Data are available under the terms of the Creative Commons Zero “No rights reserved” data waiver (CC0 1.0 Public domain dedication).

## Software availability

NIFtHool available from: https://nifthool.anvil.app/

Source code available from: https://github.com/JefferDSP/NIFTHool/tree/v1.0

Archived source code as at time of publication: https://doi.org/10.5281/zenodo.5913032. ^18^

License: CC0-1.0.
