# Supplementary material for: NIFtHool: an informatics program for identification of NifH proteins using deep neural networks
Source: F1000Res. 2022 Feb 9;11:164. [Version 1] doi: 10.12688/f1000research.107925.1 (PMC8956849; doi:10.12688/f1000research.107925.1)
Supplement: Peer review history

# Peer review: Reviewer response for version 1

**DOI:** 10.5256/f1000research.119185.r123174

**Published:** 2022-03-25

**Authors:** Lorena Isabel Barona López

**Affiliations:** 1 Artificial Intelligence and Computer Vision Research Lab, Departamento de Informática y Ciencias de la Computación (DICC), Escuela Politécnica Nacional, Quito, Ecuador

The authors have built a system that allows researchers the identification of the NifH proteins using deep learning. The paper is well written and explained clearly. Some unique suggestions would be to provide an explanation about how the number of samples was calculated, why was k-fold validation used and how were the hyperparameters determined? I think that these points will enhance the paper.

 Best regards

Are the conclusions about the tool and its performance adequately supported by the findings presented in the article?

Yes

Is the rationale for developing the new software tool clearly explained?

Yes

Is the description of the software tool technically sound?

Yes

Are sufficient details of the code, methods and analysis (if applicable) provided to allow replication of the software development and its use by others?

Yes

Is sufficient information provided to allow interpretation of the expected output datasets and any results generated using the tool?

Yes

Reviewer Expertise:

Security, Machine learning

I confirm that I have read this submission and believe that I have an appropriate level of expertise to confirm that it is of an acceptable scientific standard.

# Peer review: Reviewer response for version 1

**DOI:** 10.5256/f1000research.119185.r123112

**Published:** 2022-03-22

**Authors:** Juan Onofre Orozco-López

**Affiliations:** 1 Centro Universitario de los Lagos, Universidad de Guadalajara, Guadalajara, Mexico

**Is the rationale for developing the new software tool clearly explained?**

 The authors explained the reasons to develop this new tool, and they also is showed a summary of existing ones and their disadvantages with the current proposal. But it would be better to justify the use of this new tool from the point of view of getting extra information together at the classification capability.

 On the other hand, there are enough technical details on the requirements, the authors also widely explained the internal structure of Deep Neural Network (DNN), but didn't include information on how such a DNN is performed, additionally to the number of neurons per layer and the layers. It would be great if the authors can share or show a smaller or simpler version of such DNN, in order to replicate their experiments locally, because I found this next error while using the tool on the platform:

**" *NoServerFunctionError: No server function matching "function_pred_seq" has been registered*"**

 Maybe this error is caused by the lack of information or instructions to use the tool.

**Minor comments:**

- On page 4 it appears "NifH, nifH proteins" was written, where I understand that capital letters refer to specific chemical elements, and the lower case "n" in nifH could be a mistake.
- Figure 6 is detailed, how is the dataset divided into train or test datasets? It would be interesting to use an extra set or save an extra 10% of the training datasets to try the tool as a real test using real data. As the whole data set was used for training purposes at different iterations, the neural network has not been tested with any non-training data at any iteration. I propose to save some data sets and use them with the goal to test the performance of the neural network with unknown data by the algorithm.

Are the conclusions about the tool and its performance adequately supported by the findings presented in the article?

Yes

Is the rationale for developing the new software tool clearly explained?

Partly

Is the description of the software tool technically sound?

Yes

Are sufficient details of the code, methods and analysis (if applicable) provided to allow replication of the software development and its use by others?

Partly

Is sufficient information provided to allow interpretation of the expected output datasets and any results generated using the tool?

Yes

Reviewer Expertise:

Artificial neural networks are used mainly to develop or improve the performance of the artificial pancreas in the treatment of diabetes Mellitus type 1. Also, I used to develop automatic control algorithms, some of them using artificial neural networks

I confirm that I have read this submission and believe that I have an appropriate level of expertise to confirm that it is of an acceptable scientific standard.
